# Identifying immune cell infiltration and diagnostic biomarkers in heart failure and osteoarthritis by bioinformatics analysis

**DOI:** 10.1097/MD.0000000000034166

**Published:** 2023-06-30

**Authors:** Bo Wen, Mengna Liu, Xianyun Qin, Zhiyou Mao, Xuewei Chen

**Affiliations:** a Tianjin Institute of Environmental and Operational Medicine, Tianjin, China; b Department of Clinical Laboratory, Shanghai General Hospital, Shanghai Jiao Tong University School of Medicine, Shanghai, China; c Department of Orthopedics, No.945 Hospital of the PLA Joint Logistics Support Force, Yaan, Sichuan, China.

**Keywords:** diagnostic biomarker, GEO, heart failure, osteoarthritis, SVM model

## Abstract

Heart failure (HF) and osteoarthritis (OA) are medical conditions that can significantly impact daily activities. Evidence has shown that HF and OA may share some pathogenic mechanisms. However, the underlying genomic mechanisms remain unclear. This study aimed to explore the underlying molecular mechanism and identify diagnostic biomarkers for HF and OA. With the cutoff criteria of fold change (FC) > 1.3 and *P* < .05, 920, 1500, 2195, and 2164 differentially expressed genes (DEGs) were identified in GSE57338, GSE116250, GSE114007, and GSE169077, respectively. After making the intersection of DEGs, we obtained 90 upregulated DEGs and 51 downregulated DEGs in HF datasets and 115 upregulated DEGs and 75 downregulated DEGs in OA datasets. Afterward, we conducted genome ontology (GO) and Kyoto Encyclopedia of Genes and Genomes (KEGG) analyses, protein-protein interaction (PPI) networks, and hub genes screening based on DEGs. Then, 4 common DEGs (fibroblast activation protein alpha [FAP], secreted frizzled-related protein 4 (SFRP4), Thy-1 cell surface antigen (THY1), matrix remodeling associated 5 [MXRA5]) between HF and OA were screened and validated in GSE5406 and GSE113825 datasets, based on which we established the support vector machine (SVM) models. The combined area under the receiver operating characteristic curve (AUC) of THY1, FAP, SFRP4, and MXRA5 in the HF training and test sets reached 0.949 and 0.928. While in the OA training set and test set, the combined AUC of THY1, FAP, SFRP4, and MXRA5 reached 1 and 1, respectively. The analysis of immune cells in HF revealed high levels of dendritic cell (DC), B cells, natural killer T cell (NKT), Type 1 regulatory T cell (Tr1), cytotoxic T cell (Tc), exhausted T cell (Tex), and mucosal-associated invariant T cell (MAIT), while displaying lower levels of monocytes, macrophages, NK, CD4 + T, gamma delta T (γδ T), T helper type 1 (Th1), T helper type 2 (Th2), and effector memory T cell (Tem). Moreover, the 4 common DEGs were positively correlated with DCs and B cells and negatively correlated with γδ T. In OA patients, the abundance of monocyte, macrophage, CD4 + naïve, and natural T regulatory cell (nTreg) was higher, while the infiltration of CD8 + T, γδ T, CD8 + naïve, and MAIT was lower. The expression of THY1 and FAP was significantly correlated with macrophage, CD8 + T, nTreg, and CD8 + naïve. SFRP4 was correlated with monocyte, CD8 + T, γδ T, CD4 + naïve, nTreg, CD8 + naïve and MAIT. MXRA5 was correlated with macrophage, CD8 + T, nTreg and CD8 + naïve. FAP, THY1, MXRA5, and SFRP4 may be diagnostic biomarkers for both HF and OA, and their correlation with immune cell infiltrations suggests shared immune pathogenesis.

## 1. Introduction

Heart failure (HF) is a clinical syndrome consisting of cardinal symptoms that may be accompanied by signs (e.g., breathlessness, fatigue, elevated jugular venous pressure, pulmonary crackles, and peripheral edema). HF is a severe medical condition that can lead to higher rates of illness and death. It is characterized by abnormalities in the structure or function of the heart, which can cause high pressure within the heart and inadequate blood circulation during rest and physical exertion.^[1]^ HF is a rapidly growing public health issue with an estimated prevalence of >40 million individuals globally. In the USA, an estimated 6.2 million individuals lived with HF in 2016, and 80,480 died from HF in 2017. The etiology of HF varies according to many factors, such as age, sex, obesity, smoking, and diabetes mellitus. Most commonly, HF is due to myocardial dysfunction. Besides, valves, pericardium, endocardium, and heart rhythm abnormalities can also cause or contribute to HF.^[2–4]^

Osteoarthritis (OA) is the most common type of arthritis and a significant cause of disability in older adults. As estimated, 30.8 million adults in the US and 300 million worldwide live with OA. The burden of OA in developed countries is estimated to be between 1% and 2.5% of the gross national product.^[5,6]^ Clinically, keen OA accounts for approximately 85% of sites of OA, which involves structure alterations in the hyaline articular cartilage, subchondral bone, ligaments, capsule, synovium, and periarticular muscles. Notably, articular cartilage gets severely degenerative changes during the disease.^[7]^ Pain is the most prominent symptom suffered among OA patients. Other symptoms, joint swelling, clicking, locking, grafting, and deformity, are also present.^[8]^

Both HF and OA can significantly reduce an individual quality of life. However, current treatments for both HF and OA are limited, and their pathophysiology mechanisms are still unclear. Previous studies have shown a crosstalk between the pathogenesis of HF and OA. It was reported that about 24% of patients suffering from HF also had concurrent OA complications.^[9]^ A systematic review and meta-analysis found that individuals with OA were approximately 3 times more susceptible to HF than non-OA cohorts.^[9]^ Consistently, Swain and colleagues also confirmed that people with OA had a higher risk of developing HF in a recent study.^[10]^ It is postulated that OA and HF may share a common pathogenic mechanism. Recently, bioinformatics methods have been wildly applied in disease pathogenesis. Here, we explored the common DEGs and their correlation with immune cell infiltration in HF and OA by bioinformatic methods. Our data may guide further research in the molecular pathogenesis of HF and OA.

## 2. Materials and methods

### 2.1. Data acquisition and preprocessing

**Figure 1.**
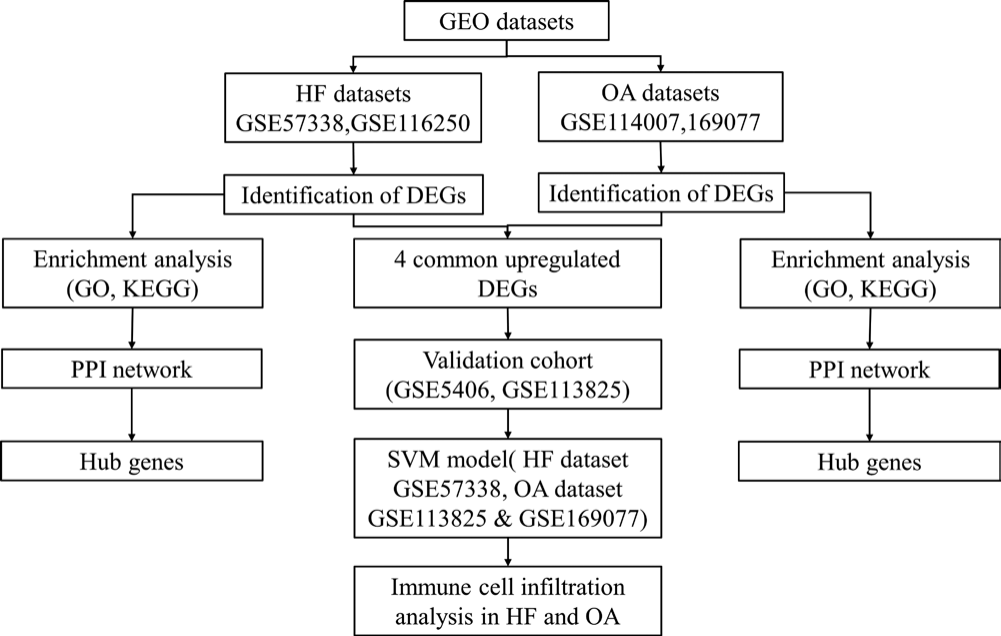
Workflow of this study. GEO = gene expression omnibus, GO = genome ontology, HF = heart failure, KEGG: Kyoto Encyclopedia of Genes and Genomes, OA = osteoarthritis, PPI = protein-protein interaction networks, SVM = support vector machine.

Gene expression profile datasets GSE114007, GSE169077, GSE57338, and GSE116250 were downloaded from the Gene Expression Omnibus database (GEO, https://www.ncbi.nlm.nih.gov/geo/) affiliated with The National Center for Biotechnology Information (https://www.ncbi.nlm.nih.gov/). This study is based on the public database, so there are no ethical issues or other conflicts of interest. GSE114007 was performed on 18 normal and 20 OA human knee cartilage tissues. GSE169077 analyzed 5 normal and 6 OA samples in cartilage tissues. GSE57338 contained 177 HF samples and 136 normal samples. In GSE116250, there were 50 HF samples and 14 normal samples. Samples were extracted from human left ventricle tissue in both GSE57338 and GSE116250. Detailed information on datasets is described in Table 1, and the workflow is shown in Figure 1. Raw microarray data were subjected to background correction, quantile normalization, and log2 transformation through the RMA algorithm of the “affy” software package. The probe ID was then converted to the gene symbol by the corresponding platform file.

**Table 1:** Detailed information on GEO datasets.

GSE number	Disease	Sample type	Organism	Disease vs control	Platform
GSE57338	Heart failure	Left ventricle	Human	177HF/136NHF	GPL11532
GSE116250	Heart failure	Left ventricle	Human	50HF/14NHF	GPL16791
GSE169077	Osteoarthritis	Knee cartilage	Human	6OA/5Normal	GPL96
GSE114007	Osteoarthritis	Knee cartilage	Human	18OA/20Normal	GPL11154
GSE5406	Heart failure	Left ventricle	Human	194HF/16NHF	GPL96
GSE113825	Osteoarthritis	Knee cartilage	Human	5OA/5Normal	GPL22516

GEO = gene expression omnibus, HF = heart failure, NHF = non-heart failing, OA = osteoarthritis.

### 2.2. Identification and visualization of differentially expressed gene (DEGs)

We evaluated the distribution of gene expression levels in different samples. Principal component analysis was applied to explore the intergroup difference and intragroup sample duplications. The statistical software R (version 4.1.1, https://www.r-project.org/) and Bioconductor packages (available online: https://www.bioconductor.org/) were applied to screen DEGs. According to the cutoff criteria of fold change (FC) > 1.3 and *P* < .05, DEGs were screened by the “limma” package.^[11]^ Visualization of DEGs was constructed by the “pheatmap” package and “ggplot2” package for the heat map and volcano map, respectively.

### 2.3. Genome ontology (GO) and Kyoto encyclopedia of genes and genomes (KEGG) pathway enrichment analyses of DEGs

The GO knowledgebase is the world largest source of information on the functions of genes, which includes biological processes (BP), cellular components (CC), and molecular functions (MF). KEGG is a database for systematically analyzing gene function. We first convert the Official Symbol of DEGs to gene IDs by “org.Hs.e.g.db” and “clusterProfiler” packages. Afterward, gene IDs were used for GO enrichment and KEGG pathway analyses. *P* < .05 was determined as a cutoff criterion for significant enrichment.

### 2.4. Protein-protein interaction (PPI) network construction and Hub gene screening

We constructed PPI networks by STRING database (https://cn.string-db.org/). Cytoscape is an open-source software wildly used in bioinformatics for visualizing molecular interaction networks.^[12]^ CytoHubba, a Cytoscape plugin, was designed to evaluate hub genes in the PPI network. According to the degree calculated by the MCC method in cytoHubba, the top 10 hub genes were screened. Nodes in the network filled with red color denoted that it has a higher degree.

### 2.5. Drawing Venn diagram

Based on the DEGs analysis, the “VennDiagram” package produced the intersection of all 4 datasets by importing upregulated and down-regulated genes.

### 2.6. Validation of the common DEGs between HF and OA

Two gene expression profiles were obtained from the GEO database: GSE5406 and GSE113825. The GSE5406 dataset, based on GPL96, contained 194 HF samples and 16 normal samples. The GSE113825 was based on GPL22516 and included 5 OA and 5 normal samples. Raw expression data were subjected to background correction, quantile normalization, and log2 transformation. We utilized the independent *t* test to evaluate the expression levels of common DEGs in both HF and OA (*P* < .05).

### 2.7. Construction of diagnostic model by support vector machine (SVM)

The SVM classifier is a supervised classification algorithm in machine learning. Integrated OA datasets were merged from GSE113825 and GSE169077. The HF dataset GSE57338 and the integrated OA dataset were randomly split into a training set (70%) and a test set (30%). The common DEGs were used to build SVM models using the “e1071” R package. Receiver operating characteristic curves were drawn, and the area under the receiver operating characteristic curve (AUC) value was determined to evaluate the diagnostic efficiency by “pROC” and “ggplot2” packages.

### 2.8. Evaluation of immune cell subtype distribution and correlations analysis with biomarkers

ImmuCellAI (Immune Cell Abundance Identifier) was performed to evaluate immune cell infiltration in HF and OA. This algorithm can calculate the abundance of immune cells, including the abundance of 18 T-cell subsets [CD4+ T cell, CD8+ T cell, CD4 + naïve, CD8 + naïve, central memory T cell, effector memory T (Tem), Type 1 regulatory T cell (Tr1), induced T regulatory cell, natural T regulatory cell (nTreg), T helper type 1 (Th1), T helper type 2 (Th2), T helper type 17, follicular Th cell, cytotoxic T cell (Tc), mucosal-associated invariant T cell (MAIT), exhausted T cell (Tex), gamma delta T (γδ T), and natural killer T (NKT) cells] and 6 other important immune cells (B cells, macrophages, monocytes, neutrophils, dendritic cell [DC], and natural killer cells) form microarray expression profiles.^[13]^ The association between biomarkers and the abundance of immune cells was evaluated using Pearson correlation analysis. All results were visualized by the “ggplot2,” “corrplot,” “ggpubr,” and “rstatix” packages.

## 3. Results

### 3.1. Identification of DEGs in HF and OA

**Figure 2.**
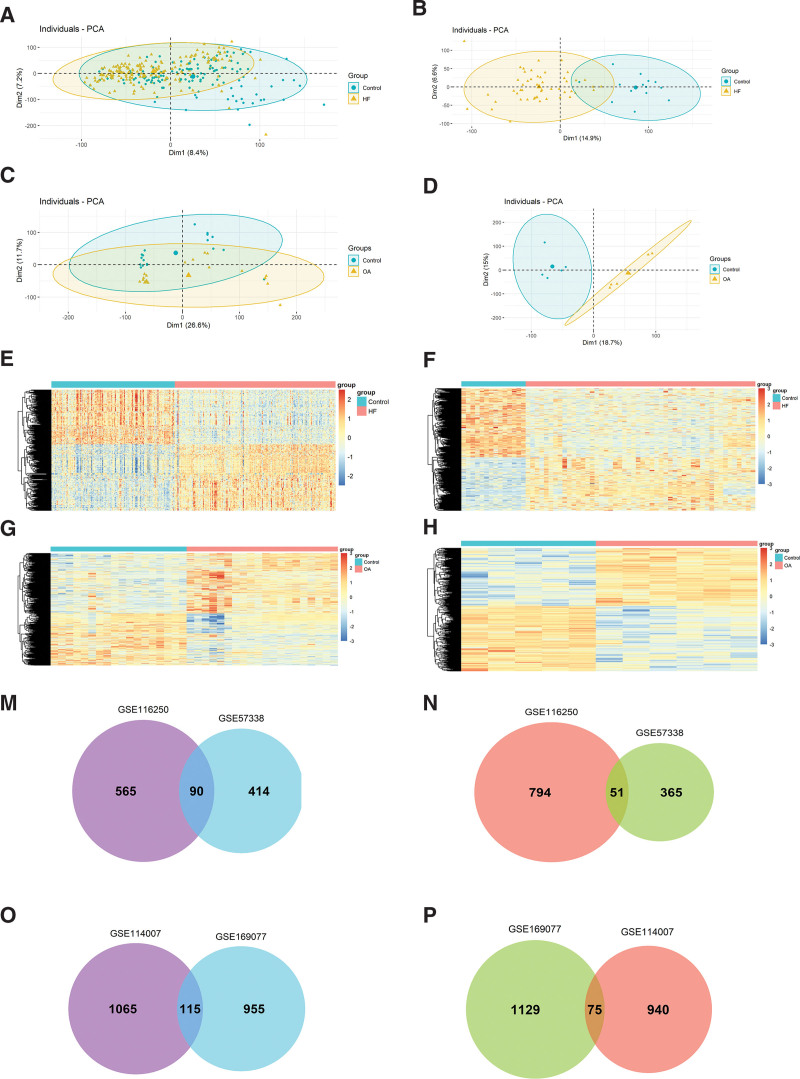
Identification of DEGs. (A–D) The principal component analysis (PCA) diagram indicates that samples in the same group were relatively uniform, and samples from different groups were significantly different (A GSE57338; B GSE116250; C GSE114007; D GSE169077). (E–H) Heatmap of DEGs (E GSE57338; F GSE116250; G GSE114007; H GSE169077). (I–L) Volcano map of DEGs. Red dots: upregulated genes. Grey dots: not DEGs. Blue dots: down-regulated genes. (I GSE57338; J GSE116250; K GSE114007; L GSE169077). (M) The Venn diagram of common upregulated DEGs in the HF datasets. (N) The Venn diagram of common down-regulated DEGs in HF datasets. (O) The Venn diagram of common upregulated DEGs in OA datasets. (P) The Venn diagram of common down-regulated DEGs in OA datasets. DEGs = differentially expressed genes, FC = fold change, HF = heart failure, OA = osteoarthritis.

Principal component analysis indicated that samples were scattered between groups and clustered within groups (Fig. 2A–D). With the cutoff criterion of *P* < .05 and FC > 1.3, we screened 920, 1500, 2195, and 2164 DEGs in GSE57338, GSE116250, GSE114007, and GSE169077, respectively. In detail, 504 upregulated genes and 416 down-regulated genes in GSE57338, 655 upregulated genes and 845 down-regulated genes in GSE116250, 1180 upregulated genes and 1015 down-regulated genes in GSE114007, 1020 upregulated genes, and 1144 down-regulated genes in GSE169077. Heat maps and volcano maps were applied to visualize the DEGs (Fig. 2E–L). A Venn diagram was conducted to reduce the false positive rate of DEGs. Finally, 141 DEGs and 190 DEGs were identified in HF and OA datasets, in which 90 and 115 genes were upregulated, and 51 and 75 were down-regulated, respectively (Fig. 2M–P).

### 3.2. Enrichment analyses of DEGs

#### 3.2.1. HF.

**Figure 3.**
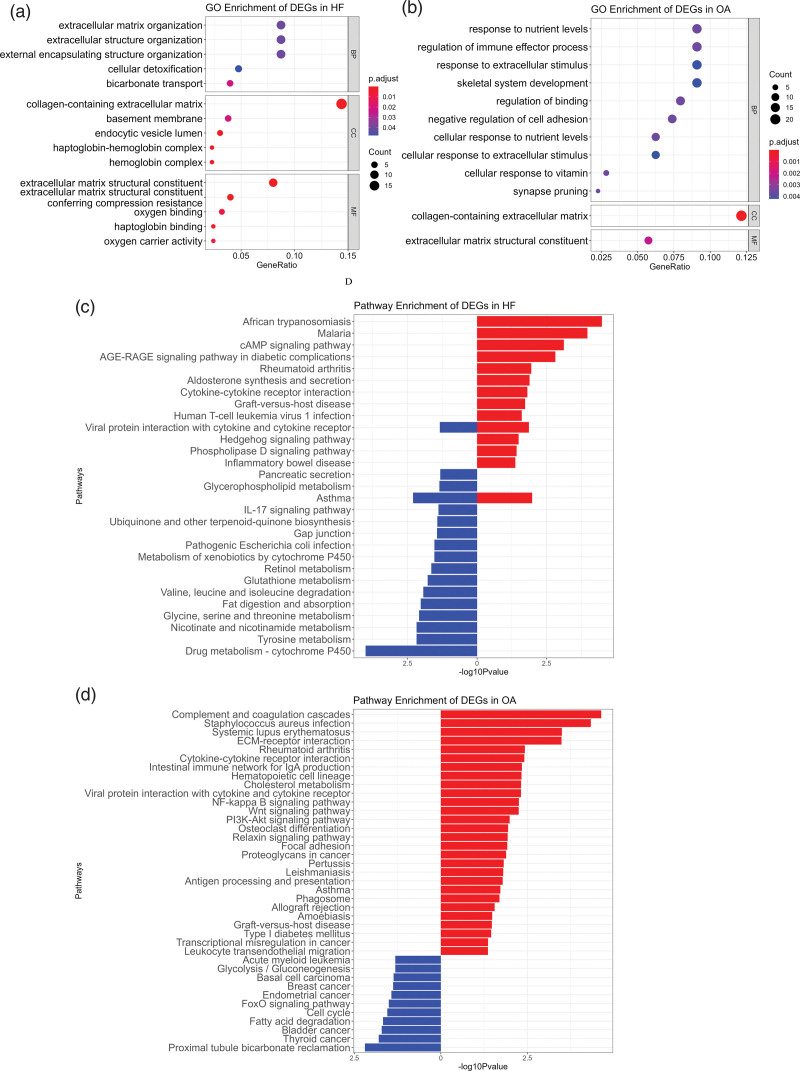
Enrichment analysis. (A) GO enrichment of common DEGs in HF datasets. (B) GO enrichment of common DEGs in OA datasets. (C) KEGG enrichment of common DEGs in HF datasets. (D) KEGG enrichment of common DEGs in OA datasets. BP = biological processes, CC = cellular component, DEGs = differentially expressed gene, GO = genome ontology, HF = heart failure, KEGG: Kyoto Encyclopedia of Genes and Genomes, MF = molecular function; OA = osteoarthritis.

As for HF, GO enrichment analysis of DEGs revealed that DEGs were considerably enriched in 6 BPs, 5 CCs, and 25 molecule functions (MFs). The BPs included extracellular matrix organization, extracellular structure organization, external encapsulating structure organization, cellular detoxification, bicarbonate transport and oxygen transport (Supplementary Table 1, http://links.lww.com/MD/J208; Fig. 3A). The CCs included collagen-containing extracellular matrix, basement membrane, endocytic vesicle lumen, haptoglobin-hemoglobin complex and hemoglobin complex (Supplementary Table 1, http://links.lww.com/MD/J208; Fig. 3A). Top 5 MFs were extracellular matrix structural constituents, extracellular matrix structural constituents conferring compression resistance, oxygen binding, haptoglobin binding and oxygen carrier activity (Supplementary Table 1, http://links.lww.com/MD/J208; Fig. 3A). The KEGG analysis indicated that the upregulated DEGs in HF enriched in 14 pathways and the top 5 of the enriched pathways were African trypanosomiasis, malaria, cAMP signaling pathway, AGE-RAGE signaling pathway, and asthma (Supplementary Table 2, http://links.lww.com/MD/J209; Fig. 3C). The top 5 KEGG pathways of downregulated DEGs were enriched in drug metabolism cytochrome P450, asthma, tyrosine metabolism, nicotinate and nicotinamide metabolism, glycine, serine and threonine metabolism (Supplementary Table 3, http://links.lww.com/MD/J210; Fig. 3C).

#### 3.2.2. OA.

GO enrichment analysis of OA DEGs revealed that DEGs were significantly enriched in 144 BPs, 1 CC, and 1 MF, respectively. The top 10 of the BPs were response to nutrient levels, regulation of immune effector process, response to extracellular stimulus, skeletal system development, regulation of binding, negative regulation of cell adhesion, cellular response to nutrient levels, cellular response to extracellular stimulus, cellular response to vitamin, synapse pruning. 1 CC was the collagen-containing extracellular matrix, and 1 MF was the extracellular matrix structural constituent (Supplementary Table 4, http://links.lww.com/MD/J211; Fig. 3B). KEGG analysis of upregulated DEGs in OA was considerably enriched in 28 pathways, and the top 5 pathways were complement and coagulation cascades, staphylococcus aureus infection, systemic lupus erythematosus, ECM-receptor interaction, rheumatoid arthritis (Supplementary Table 5, http://links.lww.com/MD/J212; Fig. 3D). The down-regulated DEGs in OA were enriched in 11 pathways, including proximal tubule bicarbonate reclamation, thyroid cancer, bladder cancer, fatty acid degradation, cell cycle, FoxO signaling pathway, endometrial cancer, breast cancer, basal cell carcinoma, glycolysis/gluconeogenesis, acute myeloid leukemia (Supplementary Table 6, http://links.lww.com/MD/J213; Fig. 3D).

### 3.3. Constructing PPI network of DEGs and screening hub genes

**Figure 4.**
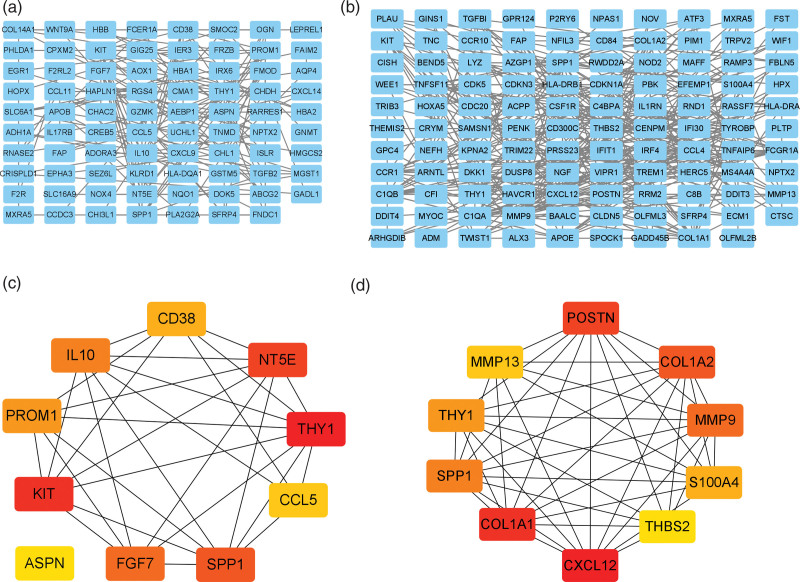
PPI network construction and hub genes screening. (A) The entire PPI network of HF DEGs. (B) The entire PPI network of OA DEGs. (C) The top 10 hub genes in the HF PPI network. (D) The top 10 hub genes in the OA PPI network. DEGs = differentially expressed gene, HF = heart failure, OA = osteoarthritis, PPI = protein-protein interaction networks.

The STRING online tool was employed to construct PPI networks of DEGs in HF and OA, respectively, which was further visualized and glorified by Cytoscape software. All 141 DEGs in HF were mapped into the original PPI network complex, which contained 141 nodes and 158 edges. Nodes disconnected from the network were deleted from the PPI network (Fig. 4A). A total of 186 DEGs of the 190 DEGs in OA filtered into the PPI network complex comprising 186 nodes and 342 edges, 4 of the 190 DEGs were not filtered into the PPI network complex (Fig. 4B). Hub genes in each PPI network was screened by cytoHubba, calculating the degrees with the MCC algorithm; the top 10 genes with the highest interaction degrees were identified as hub genes. The top 10 hub genes in HF were Thy-1 cell surface antigen (THY1), KIT, NT5E, SPP1, FGF7, IL10, PROM1, CD38, CCL5, and ASPN (Table 2 and Fig. 4C). While in the OA PPI network, the top 10 hub genes were CXCL12, COL1A1, POSTN, COL1A2, MMP9, SPP1, THY1, S100A4, MMP13 and THBS2 (Table 3 and Fig. 4D).

**Table 2:** Top 10 hub genes in HF calculated by the MCC method.

Rank	Symbol	MCC degree	Description
1	THY1	317	Thy-1 membrane glycoprotein; May play a role in cell-cell or cell-ligand interactions during synaptogenesis and other events in the brain.
2	KIT	310	V-kit Hardy-Zuckerman 4 feline sarcoma viral oncogene homolog; Tyrosine-protein kinase that acts as cell-surface receptor for the cytokine KITLG/SCF and plays an essential role in the regulation of cell survival and proliferation, hematopoiesis, stem cell maintenance, gametogenesis, mast cell development, migration and function, and in melanogenesis.
3	NT5E	290	5’-nucleotidase, ecto (CD73); Hydrolyzes extracellular nucleotides into membrane-permeable nucleosides. Exhibits AMP-, NAD-, and NMN-nucleosidase activities; Belongs to the 5’-nucleotidase family.
4	SPP1	264	Secreted phosphoprotein 1; Binds tightly to hydroxyapatite. Appears to form an integral part of the mineralized matrix. Probably important to cell-matrix interaction; Endogenous ligands.
5	FGF7	248	Heparin-binding growth factor 7; Plays an important role in the regulation of embryonic development, cell proliferation and cell differentiation. Required for normal branching morphogenesis. Growth factor active on keratinocytes. Possible major paracrine effector of normal epithelial cell proliferation;
6	IL10	195	Cytokine synthesis inhibitory factor; Inhibits the synthesis of a number of cytokines, including IFN-gamma, IL-2, IL-3, TNF and GM-CSF produced by activated macrophages and by helper T-cells; Belongs to the IL-10 family.
7	PROM1	152	Prominin-like protein 1; May play a role in cell differentiation, proliferation and apoptosis. Binds cholesterol in cholesterol- containing plasma membrane microdomains and may play a role in the organization of the apical plasma membrane in epithelial cells. During early retinal development acts as a key regulator of disk morphogenesis. Involved in regulation of MAPK and Akt signaling pathways.
8	CD38	73	2’-phospho-ADP-ribosyl cyclase/2’-phospho-cyclic-ADP-ribose transferase; Synthesizes the second messagers cyclic ADP-ribose and nicotinate-adenine dinucleotide phosphate, the former a second messenger for glucose-induced insulin secretion.
9	CCL5	35	Eosinophil chemotactic cytokine; Chemoattractant for blood monocytes, memory T-helper cells and eosinophils. Causes the release of histamine from basophils and activates eosinophils.
10	ASPN	20	Periodontal ligament-associated protein 1; Negatively regulates periodontal ligament (PDL) differentiation and mineralization to ensure that the PDL is not ossified and to maintain homeostasis of the tooth-supporting system.

HF = heart failure, THY1 = Thy-1 cell surface antigen.

**Table 3:** Top 10 hub genes in OA calculated by the MCC method.

Rank	Symbol	MCC degree	Description
1	CXCL12	22040	Pre-B cell growth-stimulating factor; Chemoattractant active on T-lymphocytes, monocytes, but not neutrophils. Activates the C-X-C chemokine receptor CXCR4 to induce a rapid and transient rise in the level of intracellular calcium ions and chemotaxis. Also binds to atypical chemokine receptor ACKR3, which activates the beta-arrestin pathway and acts as a scavenger receptor for SDF-1. SDF-1-beta (3–72) and SDF-1- alpha (3–67) show a reduced chemotactic activity.
2	COL1A1	22016	Collagen alpha-1 (I) chain; Type I collagen is a member of group I collagen (fibrillar forming collagen); Collagens
3	POSTN	21931	Periostin, osteoblast specific factor; Induces cell attachment and spreading and plays a role in cell adhesion. Enhances incorporation of BMP1 in the fibronectin matrix of connective tissues, and subsequent proteolytic activation of lysyl oxidase LOX (By similarity); Gla domain containing
4	COL1A2	21774	Collagen alpha-2 (I) chain; Type I collagen is a member of group I collagen (fibrillar forming collagen); Belongs to the fibrillar collagen family.
5	MMP9	21587	Matrix metallopeptidase 9 (gelatinase B, 92kDa gelatinase, 92kDa type IV collagenase); May play an essential role in local proteolysis of the extracellular matrix and in leukocyte migration.
6	SPP1	20343	Secreted phosphoprotein 1; Binds tightly to hydroxyapatite. Appears to form an integral part of the mineralized matrix. Probably important to cell-matrix interaction; Endogenous ligands.
7	THY1	11616	Thy-1 membrane glycoprotein; May play a role in cell-cell or cell-ligand interactions during synaptogenesis and other events in the brain; CD molecules
8	S100A4	10922	Placental calcium-binding protein; S100 calcium binding protein A4; EF-hand domain containing
9	MMP13	10104	Matrix metallopeptidase 13 (collagenase 3); Plays a role in the degradation of extracellular matrix proteins, including fibrillar collagen, fibronectin, TNC and ACAN.
10	THBS2	5931	Thrombospondin 2; Adhesive glycoprotein that mediates cell-to-cell and cell-to-matrix interactions. Ligand for CD36 mediating antiangiogenic properties.

MMPs = matrix metalloproteinases, OA = osteoarthritis, THY1 = Thy-1 cell surface antigen.

### 3.4. Validation of common DEGs between HF and OA

**Figure 5.**
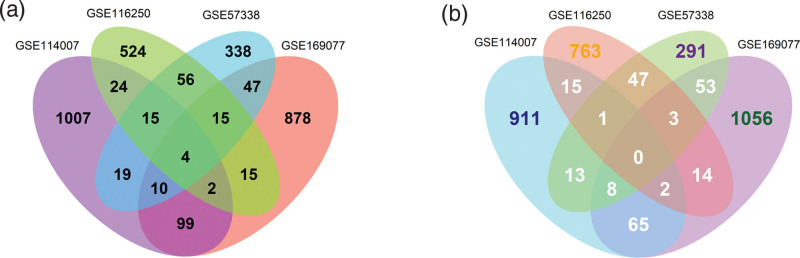
Venn diagram of common DEGs between HF and OA datasets. (A) The Venn diagram of common upregulated expressed genes in HF and OA datasets. (B) The Venn diagram of common downregulated expressed genes in HF and OA datasets. DEGs = differentially expressed gene, HF = heart failure, OA = osteoarthritis.

**Figure 6.**
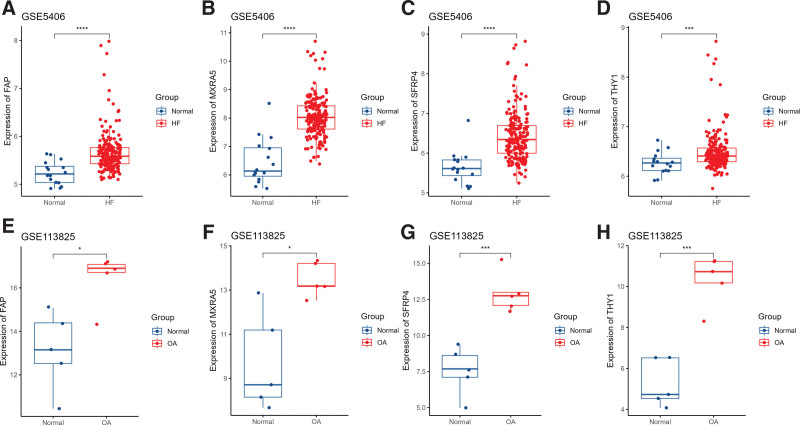
Validation of the 4 common DEGs in GEO datasets. (A–D) FAP, MXRA5, SFRP4, and THY1 expression were significantly higher in HF patients than in healthy individuals in the GSE5406 dataset. (E–H) Expression of FAP, MXRA5, SFRP4, and THY1 was significantly higher in OA patients than in healthy individuals in the GSE113825 dataset (*P < .05, **P < .01, ***P < .001, ****P < .0001). DEGs = differentially expressed gene, FAP = fibroblast activation protein alpha, GEO = gene expression omnibus, HF = heart failure, MXRA5 = matrix remodeling associated 5, OA = osteoarthritis, SFRP4 = secreted frizzled-related protein 4, THY1 = Thy-1 cell surface antigen.

As shown in Figure 5, four common upregulated genes between HF and OA were screened: fibroblast activation protein alpha (FAP), THY1, matrix remodeling associated 5 (MXRA5), and secreted frizzled-related protein 4 (SFRP4). (Table 4 and Fig. 5A and B). We further verified the expression of common upregulated genes in another 2 external GEO datasets (GSE5406 and GSE113825) (Fig. 6). Statistical significance for the *t* test was *P* < .05.

**Table 4:** Common upregulated differentially expressed genes.

Gene symbol	Description	GSE57338		GSE116250		GSE114007		GSE169077	
		logFC	*P* value	logFC	*P* value	logFC	*P* value	logFC	*P* value
SFRP4	Secreted frizzled-related protein 4	1.78	4.92E-45	1.19	8.57E-05	1.75	6.81E-06	0.86	.003369
MXRA5	Matrix remodeling associated 5	1.23	1.91E-32	0.97	9.69E-07	0.53	3.64E-06	2.87	4.36E-06
FAP	Fibroblast activation protein alpha	0.60	1.22E-11	0.62	.000138	0.42	2.05E-08	1.97	1.73E-06
THY1	Thy-1 cell surface antigen	0.41	2.15E-08	0.49	.015453	1.17	1.03E-06	1.67	1.48E-05

FAP = fibroblast activation protein alpha, FC = fold change, MXRA5 = matrix remodeling associated 5, SFRP4 = secreted frizzled-related protein 4, THY1 = Thy-1 cell surface antigen.

### 3.5. Diagnostic model for HF and OA

**Figure 7.**
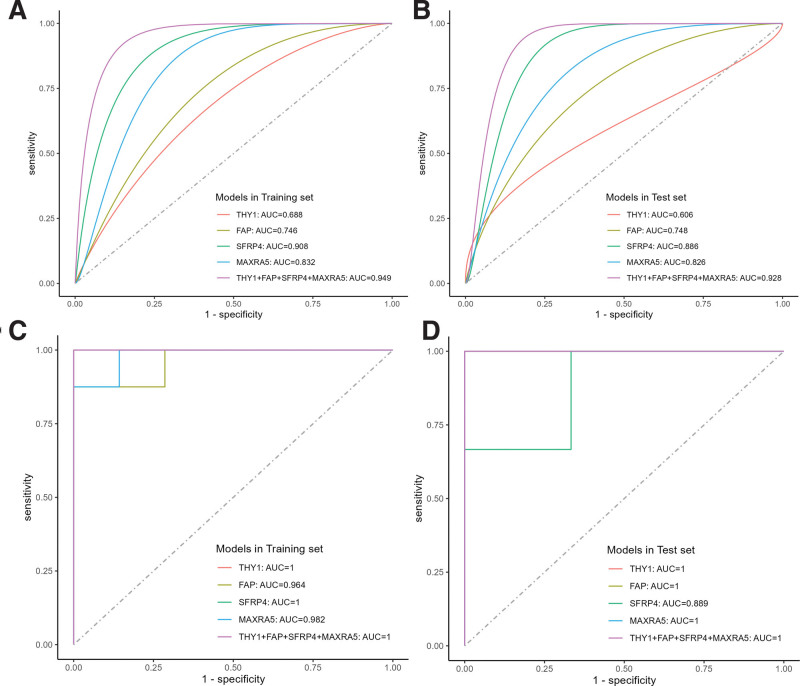
Diagnostic effectiveness of the SVM model for HF and OA. (A) ROC analysis of the SVM models based on THY1, FAP, SFRP4, and MXRA5 in the HF training set. (B) ROC analysis of the SVM models based on THY1, FAP, SFRP4, and MXRA5 in the HF test set. (C) ROC analysis of the SVM models based on THY1, FAP, SFRP4, and MXRA5 in the OA training set. (D) ROC analysis of the SVM models based on THY1, FAP, SFRP4, and MXRA5 in the OA test set. FAP = fibroblast activation protein alpha, HF = heart failure, MXRA5 = matrix remodeling associated 5, OA = osteoarthritis, ROC = receiver operating characteristic curve, SFRP4 = secreted frizzled-related protein 4, SVM = support vector machine, THY1 = Thy-1 cell surface antigen.

We randomly divided all samples in HF datasets GSE57338 and the integrated OA dataset into a training set (70%) and a test set (30%). SVM models were constructed by the “e1071” R package in the training set using the 4 biomarkers. As shown in Figure 7A and B, the AUC value of THY1, FAP, SFRP4, and MXRA5 were 0.688, 0.746, 0.908, 0.832, respectively, in the HF training set, while in the HF test set, they were 0.606, 0.748, 0.886, 0.826. The combined AUC of THY1, FAP, SFRP4, and MXRA5 in the HF training and test sets reached 0.949 and 0.928. As shown in Figure 7C and D, the AUC value of THY1, FAP, SFRP4, and MXRA5 were 1, 0.964, 1, 0.982, respectively, in the OA training set, while in the OA test set, they were 1, 1, 0.889, 1. The combined AUC of THY1, FAP, SFRP4, and MXRA5 reached 1 and 1 in the OA training and test sets. THY1, FAP, SFRP4, and MXRA5 have demonstrated exceptional specificity and sensitivity in diagnosing HF and OA.

### 3.6. Immune cell infiltration analysis and correlation with biomarkers in HF and OA

**Figure 8.**
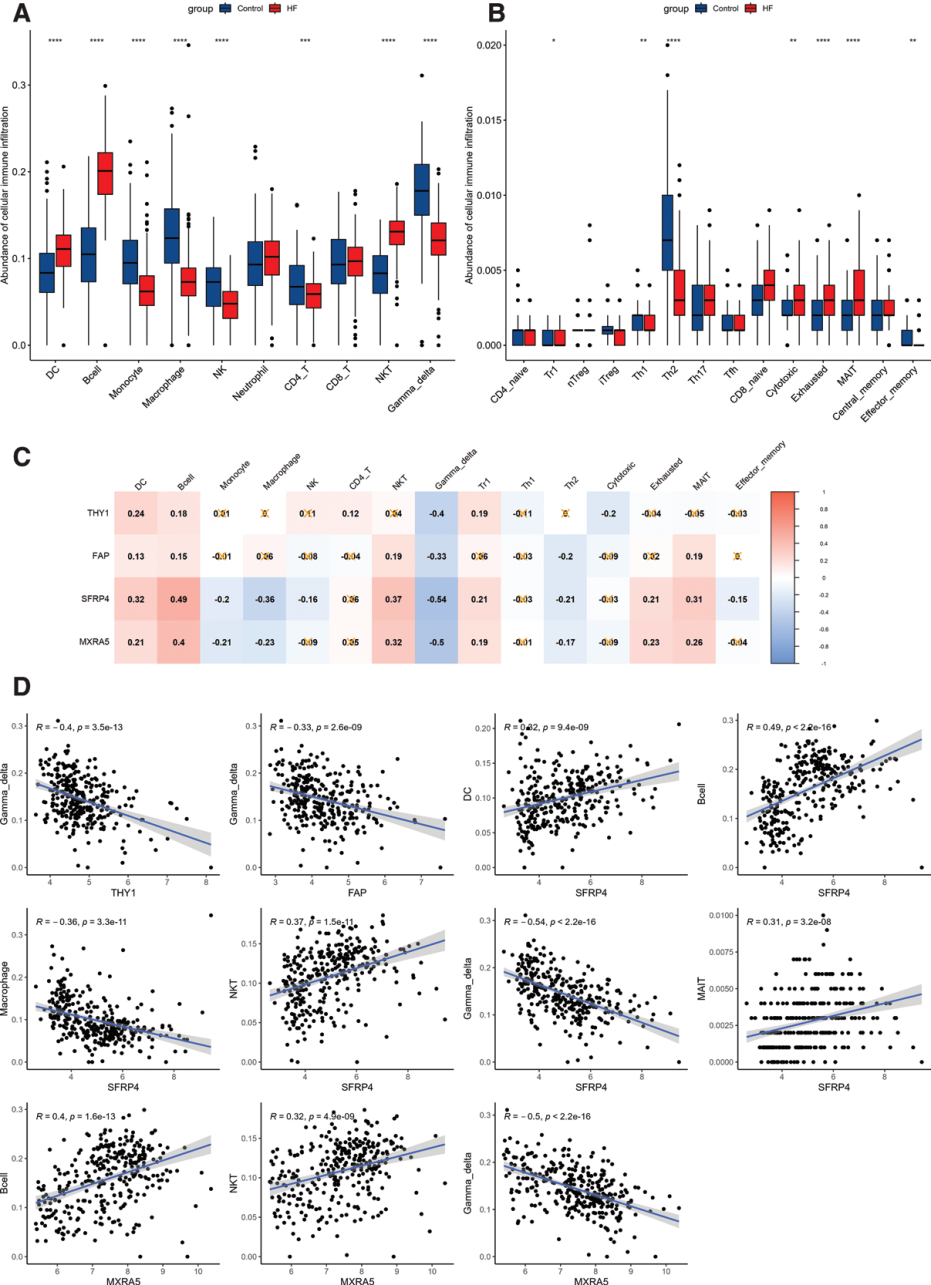
Immune cell infiltration evaluation and correlation analysis with biomarkers in HF dataset. (A and B) Immune cell abundance between HF and normal tissues in GSE57338. (C) Correlation among differential immune cell abundance and 4 biomarkers. (D) Pearson test results of significant immune cell infiltration and biomarkers (*P < .05, **P < .01, ***P < .001, ****P < .0001). HF = heart failure.

**Figure 9.**
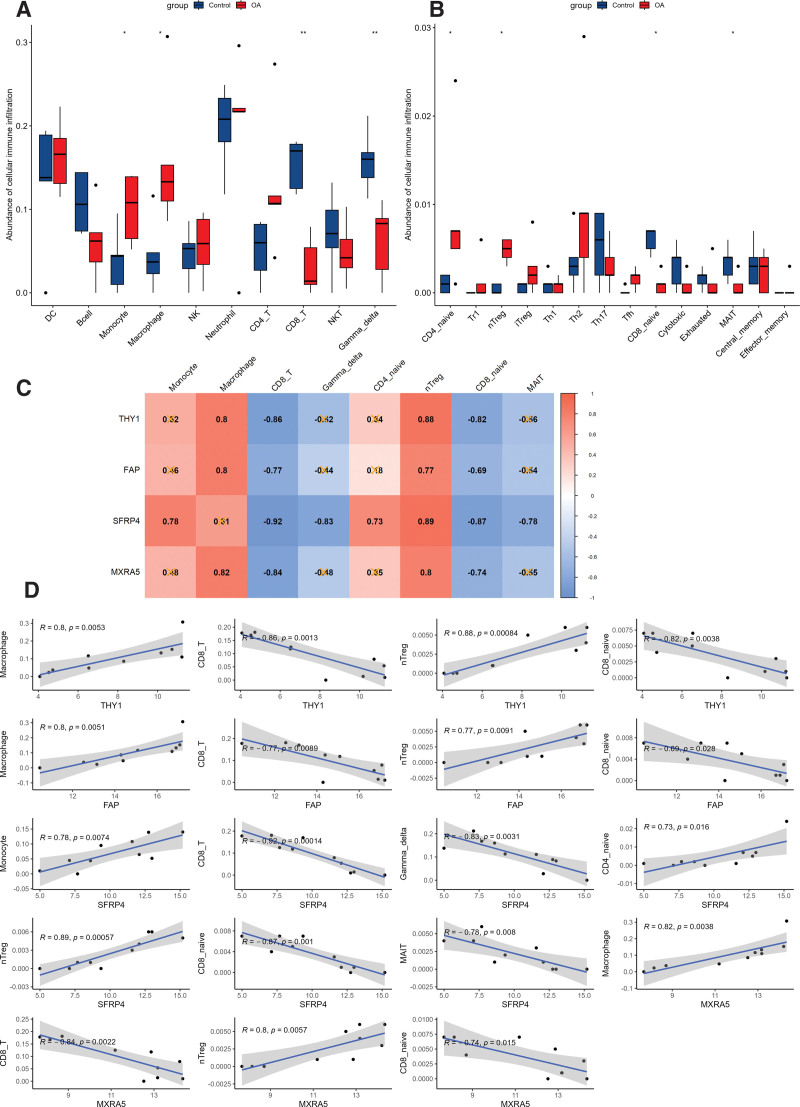
Immune cell infiltration evaluation and correlation analysis with biomarkers in OA dataset. (A and B) Immune cell abundance between OA and normal tissues in GSE113825. (C) Correlation among differential immune cell abundance and 4 biomarkers. (D) Pearson test results of significant immune cell infiltration and biomarkers (*: *P* < .05, **: *P* < .01, ***: *P* < .001, ****: *P* < .0001). OA = osteoarthritis.

The abundance of 24 types of immune cells between HF and normal left ventricular tissue in the GSE57338 dataset was compared by independent *t* test (*P* < .05). As shown in Figure 8A and B, the abundance of DC, B cells, monocytes, macrophages, natural killer cells, CD4 + T cells, NKT cells, γδ T cells, Tr1 cells, Th1 cells, Th2 cells, Tc cells, Tex cells, MAIT cells, and Tem cells were significantly different. The correlation between immune cells and biomarkers was then evaluated (Fig. 8C). The results of the Pearson test were shown in Figure 8D, with the cutoff criteria *P* < .05 and correlation coefficient > 0.3, 11 types of immune cells were significantly related to the 4 biomarkers. In the same way, the immune cell infiltration abundance in the cartilage of OA and control in the GSE113825 dataset were analyzed. The significant abundance of immune cells were monocytes, macrophages, CD8 + T cells, γδ T cells, CD4 + naïve cells, nTreg cells, CD8 + naïve cells, and MAIT cells (Fig. 9A and B). The correlation between biomarkers and immune cell abundance was calculated and visualized in Figure 9C. The Pearson test results were presented in Figure 9D.

## 4. Discussion

Patient quality of life is significantly affected by HF and OA, leading to a substantial social and economic burden. Although numerous basic and clinical research has been carried out, the molecular mechanisms of HF and OA remain to be elucidated. Cumulative evidence has indicated that HF and OA may be associated, and it is assumed that HF and OA are likely to share a common pathogenesis and correlative gene susceptibility.^[9,10,14]^ However, the correlations between the 2 diseases at the gene level have yet to be reported. Gene microarray technology and high-throughput sequencing are 2 primary methods used in gene expression research, which have been wildly used to predict the potential diagnostic and therapeutic targets for HF and OA.

In this study, all 6 datasets downloaded from the GEO database were grouped into training datasets (GSE57338, GSE116250, GSE114007, GSE169077) and validation datasets (GSE5406, GSE113825). With the cutoff criteria of FC > 1.3 and *P* < .05, 920, 1500, 2195, and 2164 DEGs were screened in GSE57338, GSE116250, GSE114007, and GSE169077, respectively. Common DEGs identified in GSE57338 and GSE116250 included 90 upregulated DEGs and 51 down-regulated DEGs in HF. While for OA, the common DEGs were selected in GSE114007 and GSE169077, including 115 upregulated DEGs and 75 down-regulated DEGs. GO enrichment analysis and KEGG enrichment analysis were carried out based on DEGs. The HF DEGs were mainly enriched in functions related to extracellular matrix organization, extracellular structure organization, external encapsulating structure organization, cellular detoxification, bicarbonate transport, and oxygen transport. A proteomic study by Su et al showed that carbonic anhydrase 2 (CA2) and 3 (CA3), critical in regulating bicarbonate transport, were risk factors and potential biomarkers for dilated cardiomyopathy-associated HF.^[15]^ As reviewed, dynamic extracellular matrix changes are closely related to the pathogenesis of HF.^[16,17]^ The top 5 HF pathways enriched in upregulated DEGs were African trypanosomiasis, malaria, cAMP signaling, AGE-RAGE signaling, and asthma. The top 5 KEGG pathways enriched in HF downregulated DEGs were drug metabolism cytochrome P450, asthma, tyrosine metabolism, nicotinate and nicotinamide metabolism, glycine, serine, and threonine metabolism.

GO enrichment analysis of OA DEGs was significantly enriched in 144 BPs, 1 CC, and 1 MF, respectively. The top 5 BPs were response to nutrient levels, regulation of immune effector process, response to extracellular stimulus, skeletal system development, and regulation of binding. The top 5 KEGG pathways enriched in OA upregulated DEGs were complement and coagulation cascades, staphylococcus aureus infection, systemic lupus erythematosus, ECM-receptor interaction, and rheumatoid arthritis. The top 5 KEGG pathways enriched in OA downregulated DEGs were proximal tubule bicarbonate reclamation, thyroid cancer, bladder cancer, cell cycle, and FoxO signaling pathway.

By CytoHubba, the top 10 hub genes were screened in the PPI network. As for HF, top 10 hub genes were THY1, KIT, NT5E, SPP1, FGF7, IL10, PROM1, CD38, CCL5, and ASPN. While in the OA PPI network, hub genes were CXCL12, COL1A1, POSTN, COL1A2, MMP9, SPP1, THY1, S100A4, MMP13, and THBS2. Notably, THY1 was selected as the hub gene in HF and OA PPI networks, indicating that THY1 may play a critical role in the pathogenesis of HF and OA.

Subsequently, 4 common DEGs were identified from all 4 training datasets and further validated in the validation datasets (FAP, THY1, MXRA5, SFRP4). Consistently, the gene levels of the 4 genes were significantly higher in HF and OA patients compared with normal individuals.

Next, we established diagnostic models for HF and OA with the SVM method using the 4 common DEGs, which had excellent specificity and sensitivity. Finally, we evaluated the immune cell infiltration and its correlation with biomarkers. Our results indicate that biomarkers are significantly correlated with immune cell abundance.

FAP is an emerging therapeutic target due to its upregulated expression in tumor stroma and tissue remodeling, including liver fibrosis, lung fibrosis, colorectal cancer, ovarian cancer, prostate cancer, and arthritis.^[18–21]^ Besides, FAP has been associated with cell migration and cell invasiveness. Fang et al reported that FAP overexpression in solid tumor tissue was significantly associated with poor overall survival and tumor progression.^[22]^ Conformably, FAP was supposed to be significantly associated with tumor size and clinical stage in osteosarcoma.^[23]^ It has also been predicted to play a novel role in the attachment of synovial fibroblasts to cartilage, which further contributes to proteoglycan loss and cartilage degradation.^[24]^ THY1 also known as CD90, is widely regarded as a cell surface marker for sorting a subpopulation of fibroblasts in the heart. Lack of THY1 expression in ventricular fibroblasts leads to cardiomyocyte contractile dysfunction and tissue fibrosis.^[25]^ On the contrary, upregulated THY1 expression may play a protective role in HF. Increased expression of THY1 in epithelial ovarian cancer predicts poor prognosis with increased proliferative and self-renewal capacity.^[26]^ The altered expression of THY1 is also related to poor prognosis in pancreatic ductal adenocarcinoma and acts as an anchor for monocyte and macrophage.^[27]^ MXRA5 was identified as co-upregulated DEGs in 3 GEO datasets of ischemic cardiomyopathy and validated by qRT-PCR.^[28]^ Zhou and colleagues have consistently reported that MXRA5 and SFRP4 were significantly upregulated in HF tissue, and MXRA5 provided a clear picture of the prognosis of patients with HF.^[29]^ Furthermore, MXRA5 has always been found in injured tissue and functions as an anti-inflammatory and anti-fibrotic molecule.^[30]^ In addition, the upregulation of MXRA5 largely contributes to developing benign prostatic hyperplasia through increasing cell proliferation via the MAPK pathway.^[31]^ Yi et al identified MXRA5 as a biomarker for non-small cell lung cancer, and high MXRA5 expression was correlated with tumor progression and overall survival.^[32]^ In another research, MXRA5 was an independent indicator of poor prognosis in glioma patients, and its expression was highly correlated with macrophage infiltration.^[33]^ SFRP4, is a member of the SFRPs family, participating in various pathogenesis by regulating the Wnt signal transduction system.^[34]^ James et al reported an increased SFRP4 gene expression in chondrocytes of human OA cartilage, which was predicted to be an anti-apoptotic factor.^[35]^ Additionally, increased SFRP4 is associated with developing gestational diabetes mellitus and polycystic ovary syndrome.^[36,37]^

In immune cell infiltration analysis, DC, B cell, NKT, Tr1, Tc, Tex, and MAIT were highly infiltrated in HF. At the same time, the abundance of monocyte, macrophage, NK, CD4 + T, γδ T, Th1, Th2, and Tem was lower, indicating their essential roles in the etiology of HF. Moreover, we found that the expression of the 4 common DEGs was significantly positively correlated with DCs and B cells and negatively correlated with γδ T. Besides, SFRP4 was also significantly positively correlated with NKT and MAIT, and MXRA5 was significantly positively correlated with NKT.

In OA cartilage, abundance of monocyte, macrophage, CD4 + naïve and nTreg is higher, while the infiltration of CD8 + T, γδ T, CD8 + naïve and MAIT were lower. The expression of THY1 and FAP was significantly correlated with macrophage, CD8 + T, nTreg and CD8 + naïve. SFRP4 was correlated with monocyte, CD8 + T, γδ T, CD4 + naïve, nTreg, CD8 + naïve and MAIT. MXRA5 was correlated with macrophage, CD8 + T, nTreg and CD8 + naïve.

Myocardial infarction is one of the most common causes of HF. After the ischemic injury to the myocardium, DC responds to cardiomyocyte necrosis, presenting cardiac antigen to T cells and potentially leading to an excessive autoimmune response against the heart. Elvira et al indicated that myocardial injury led to local infiltration and activation of cross-priming DCs, further activating Tc cells infiltrating the heart.^[38]^ Yusuke and colleagues found that circulating myeloid DC and plasmacytoid DC were reduced, and the reduction would be restored after treatment.^[39]^ B cells have 3 main functions: antibody production, antigen presentation, and cytokine production. Evidence suggests that B cells play a crucial role in HF with reduced ejection fraction via antibody and cytokine secretion.^[40]^ The recruitment of B cells is significantly high from day 1 and remains high even after 14 days post-myocardial infraction.^[41]^ B cell activation may further enhance the activation of the immune system and adverse cardiac changes, including fibrosis, hypertrophy, and contractile dysfunction.

Synovial and articular inflammatory environments contribute significantly to OA progression. Macrophages are responsible for innate immunity and exhibit high plasticity. After initial stimulation, macrophages acquire a phenotype ranging from pro-inflammatory (M1) to anti-inflammatory (M2).^[42]^ In a recent review, Wu et al introduced that synovial macrophage could secrete pro-inflammation signal molecules such as IL-1 and TNF-α and activate the production of matrix metalloproteinases. These matrix metalloproteinases and inflammatory mediators stimulated the upregulation of ECM-degradative enzymes and further aggravated cartilage matrix destruction.^[43]^ Our results demonstrated that macrophage infiltration was significantly high in OA, perhaps indicating that M1 functioned as a critical factor in OA onset. Consistently, macrophages and monocyte have been detected in the synovial fluid of OA joints, which positively correlate with joint stiffness, pain, and reduced quality of life.^[44]^ Harini et al demonstrated that monocyte recruitment via CCL2/CCR2 signal propagated inflammation and tissue damage in OA.^[45]^ Therefore, our study estimated that the 4 common DEGs were associated with the occurrence and progress of OA and HF via regulating several immune cells.

However, some limitations to our study should be noted. Firstly, our results are based on a public database and computational algorithm. Besides, the sample size is not large enough. Further validations in large-scale clinical trials are required. Secondly, our data merely support the correlation between the 4 common DEGs and immunocytes in HF and OA rather than revealing the causality.

## 5. Conclusion

FAP, THY1, MXRA5, and SFRP4 can be selected as diagnostic biomarkers for both HF and OA, and they are significantly correlated with immune cell infiltration, indicating HF and OA may share the immune pathogenesis. Monocytes, macrophages, γδ T, and MAIT may be potential immunotherapy targets in HF and OA.

## Author contributions

**Conceptualization:** Xianyun Qin, Zhiyou Mao.

**Data curation:** Bo Wen, Mengna Liu.

**Formal analysis:** Bo Wen, Mengna Liu.

**Supervision:** Xuewei Chen.

**Visualization:** Bo Wen.

**Writing – original draft:** Bo Wen, Mengna Liu.

**Writing – review & editing:** Xuewei Chen.

## Supplementary Material

## References

[1] McDonagh TA Metra M Adamo M. 2021 ESC Guidelines for the diagnosis and treatment of acute and chronic heart failure. Eur Heart J. 2021;42:3599–726. 34447992 10.1093/eurheartj/ehab368

[2] Baman JR Ahmad FS. Heart failure. JAMA. 2020;324:1015. 32749448 10.1001/jama.2020.13310

[3] Virani SS Alonso A Benjamin EJ. Heart disease and stroke statistics-2020 update: a report from the American Heart Association. Circulation. 2020;141:e139–596. 31992061 10.1161/CIR.0000000000000757

[4] Ziaeian B Fonarow GC. Epidemiology and aetiology of heart failure. Nat Rev Cardiol. 2016;13:368–78. 26935038 10.1038/nrcardio.2016.25 PMC4868779

[5] Hunter DJ Schofield D Callander E. The individual and socioeconomic impact of osteoarthritis. Nat Rev Rheumatol. 2014;10:437–41. 24662640 10.1038/nrrheum.2014.44

[6] O’Neill TW McCabe PS McBeth J. Update on the epidemiology, risk factors and disease outcomes of osteoarthritis. Best Pract Res Clin Rheumatol. 2018;32:312–26. 30527434 10.1016/j.berh.2018.10.007

[7] Hunter DJ Bierma-Zeinstra S. Osteoarthritis. Lancet. 2019;393:1745–59. 31034380 10.1016/S0140-6736(19)30417-9

[8] Abramoff B Caldera FE. Osteoarthritis: pathology, diagnosis, and treatment options. Med Clin North Am. 2020;104:293–311. 32035570 10.1016/j.mcna.2019.10.007

[9] Prior JA Jordan KP Kadam UT. Associations between cardiovascular disease severity, osteoarthritis co-morbidity and physical health: a population-based study. Rheumatology (Oxford). 2014;53:1794–802. 24821851 10.1093/rheumatology/keu175

[10] Swain S Coupland C Mallen C. Temporal relationship between osteoarthritis and comorbidities: a combined case control and cohort study in the UK primary care setting. Rheumatology (Oxford). 2021;60:4327–39. 33506862 10.1093/rheumatology/keab067 PMC8410005

[11] Ritchie ME Phipson B Wu D. limma powers differential expression analyses for RNA-sequencing and microarray studies. Nucleic Acids Res. 2015;43:e47. 25605792 10.1093/nar/gkv007 PMC4402510

[12] Shannon P Markiel A Ozier O. Cytoscape: a software environment for integrated models of biomolecular interaction networks. Genome Res. 2003;13:2498–504. 14597658 10.1101/gr.1239303 PMC403769

[13] Miao YR Zhang Q Lei Q. ImmuCellAI: a unique method for comprehensive T-cell subsets abundance prediction and its application in cancer immunotherapy. Adv Sci (Weinh). 2020;7:1902880. 32274301 10.1002/advs.201902880 PMC7141005

[14] Hall AJ Stubbs B Mamas MA. Association between osteoarthritis and cardiovascular disease: systematic review and meta-analysis. Eur J Prev Cardiol. 2015;23:938–46. 26464295 10.1177/2047487315610663

[15] Su H Hu K Liu Z. Carbonic anhydrase 2 and 3 as risk biomarkers for dilated cardiomyopathy associated heart failure. Ann Palliat Med. 2021;10:12554–65. 35016406 10.21037/apm-21-3561

[16] Frangogiannis NG. The extracellular matrix in myocardial injury, repair, and remodeling. J Clin Invest. 2017;127:1600–12. 28459429 10.1172/JCI87491 PMC5409799

[17] Frangogiannis NG. The extracellular matrix in ischemic and nonischemic heart failure. Circ Res. 2019;125:117–46. 31219741 10.1161/CIRCRESAHA.119.311148 PMC6588179

[18] Hamson EJ Keane FM Tholen S. Understanding fibroblast activation protein (FAP): substrates, activities, expression and targeting for cancer therapy. Proteomics Clin Appl. 2014;8:454–63. 24470260 10.1002/prca.201300095

[19] Coto-Llerena M Ercan C Kancherla V. High expression of FAP in colorectal cancer is associated with angiogenesis and immunoregulation processes. Front Oncol. 2020;10:979. 32733792 10.3389/fonc.2020.00979 PMC7362758

[20] Li M Cheng X Rong R. High expression of fibroblast activation protein (FAP) predicts poor outcome in high-grade serous ovarian cancer. BMC Cancer. 2020;20:1032. 33109151 10.1186/s12885-020-07541-6 PMC7590670

[21] Vlachostergios PJ Karathanasis A Tzortzis V. Expression of fibroblast activation protein is enriched in neuroendocrine prostate cancer and predicts worse survival. Genes (Basel). 2022;13:135. 35052475 10.3390/genes13010135 PMC8774973

[22] Green J Liu F Qi L. Fibroblast activation protein overexpression and clinical implications in solid tumors: a meta-analysis. PLoS One. 2015;10:e0116683. 25775399 10.1371/journal.pone.0116683 PMC4361589

[23] Zhang L Yang L Xia ZW. The role of fibroblast activation protein in progression and development of osteosarcoma cells. Clin Exp Med. 2020;20:121–30. 31745677 10.1007/s10238-019-00591-6

[24] Alimadadi A Aryal S Manandhar I. Identification of upstream transcriptional regulators of ischemic cardiomyopathy using cardiac RNA-Seq meta-analysis. Int J Mol Sci. 2020;21:3472. 32423033 10.3390/ijms21103472 PMC7278960

[25] Li Y Song D Mao L. Lack of Thy1 defines a pathogenic fraction of cardiac fibroblasts in heart failure. Biomaterials. 2020;236:119824. 32028169 10.1016/j.biomaterials.2020.119824 PMC7024042

[26] Connor EV Saygin C Braley C. Thy-1 predicts poor prognosis and is associated with self-renewal in ovarian cancer. J Ovarian Res. 2019;12:112. 31735168 10.1186/s13048-019-0590-5 PMC6858973

[27] Shi J Lu P Shen W. CD90 highly expressed population harbors a stemness signature and creates an immunosuppressive niche in pancreatic cancer. Cancer Lett. 2019;453:158–69. 30954649 10.1016/j.canlet.2019.03.051

[28] Cao J Liu Z Liu J. Bioinformatics analysis and identification of genes and pathways in ischemic cardiomyopathy. Int J Gen Med. 2021;14:5927–37. 34584445 10.2147/IJGM.S329980 PMC8464396

[29] Zhou J Zhang W Wei C. Weighted correlation network bioinformatics uncovers a key molecular biosignature driving the left-sided heart failure. BMC Med Genomics. 2020;13:93. 32620106 10.1186/s12920-020-00750-9 PMC7333416

[30] Poveda J Sanz AB Fernandez-Fernandez B. MXRA5 is a TGF-beta1-regulated human protein with anti-inflammatory and anti-fibrotic properties. J Cell Mol Med. 2017;21:154–64. 27599751 10.1111/jcmm.12953 PMC5192817

[31] Xiao H Jiang Y He W. Identification and functional activity of matrix-remodeling associated 5 (MXRA5) in benign hyperplastic prostate. Aging (Albany NY). 2020;12:8605–21. 32392178 10.18632/aging.103175 PMC7244086

[32] He Y Chen X Liu H. Matrix-remodeling associated 5 as a novel tissue biomarker predicts poor prognosis in non-small cell lung cancers. Cancer Biomark. 2015;15:645–51. 26406953 10.3233/CBM-150504 PMC12965440

[33] Sun JZ Zhang JH Li JB. MXRA5 is a novel immune-related biomarker that predicts poor prognosis in glioma. Dis Markers. 2021;2021:6680883. 34211612 10.1155/2021/6680883 PMC8211501

[34] Pawar NM Rao P. Secreted frizzled related protein 4 (sFRP4) update: a brief review. Cell Signal. 2018;45:63–70. 29360572 10.1016/j.cellsig.2018.01.019

[35] James IE Kumar S Barnes MR. FrzB-2: a human secreted frizzled-related protein with a potential role in chondrocyte apoptosis. Osteoarthritis Cartilage. 2000;8:452–63. 11069730 10.1053/joca.1999.0321

[36] Yuan XS Zhang M Wang HY. Increased secreted frizzled-related protein 4 and ficolin-3 levels in gestational diabetes mellitus women. Endocr J. 2018;65:499–508. 29491225 10.1507/endocrj.EJ17-0508

[37] Bicer M Alarslan P Guler A. Elevated circulating levels of secreted frizzled-related protein 4 in relation to insulin resistance and androgens in women with polycystic ovary syndrome. J Endocrinol Invest. 2020;43:305–13. 31486991 10.1007/s40618-019-01108-4

[38] Forte E Perkins B Sintou A. Cross-priming dendritic cells exacerbate immunopathology after ischemic tissue damage in the heart. Circulation. 2021;143:821–36. 33297741 10.1161/CIRCULATIONAHA.120.044581 PMC7899721

[39] Sugi Y Yasukawa H Kai H. Reduction and activation of circulating dendritic cells in patients with decompensated heart failure. Int J Cardiol. 2011;147:258–64. 19923020 10.1016/j.ijcard.2009.09.524

[40] Bermea K Bhalodia A Huff A. The role of B cells in cardiomyopathy and heart failure. Curr Cardiol Rep. 2022;24:935–46. 35689723 10.1007/s11886-022-01722-4 PMC9422953

[41] Yan X Anzai A Katsumata Y. Temporal dynamics of cardiac immune cell accumulation following acute myocardial infarction. J Mol Cell Cardiol. 2013;62:24–35. 23644221 10.1016/j.yjmcc.2013.04.023

[42] Yunna C Mengru H Lei W. Macrophage M1/M2 polarization. Eur J Pharmacol. 2020;877:173090. 32234529 10.1016/j.ejphar.2020.173090

[43] Wu CL Harasymowicz NS Klimak MA. The role of macrophages in osteoarthritis and cartilage repair. Osteoarthritis Cartilage. 2020;28:544–54. 31926267 10.1016/j.joca.2019.12.007 PMC7214213

[44] Gomez-Aristizabal A Gandhi R Mahomed NN. Synovial fluid monocyte/macrophage subsets and their correlation to patient-reported outcomes in osteoarthritic patients: a cohort study. Arthritis Res Ther. 2019;21:26. 30658702 10.1186/s13075-018-1798-2 PMC6339358

[45] Raghu H Lepus CM Wang Q. CCL2/CCR2, but not CCL5/CCR5, mediates monocyte recruitment, inflammation and cartilage destruction in osteoarthritis. Ann Rheum Dis. 2017;76:914–22. 27965260 10.1136/annrheumdis-2016-210426 PMC5834918

