# Supplementary material for: Identifying immune cell infiltration and diagnostic biomarkers in heart failure and osteoarthritis by bioinformatics analysis
Source: Medicine (Baltimore). 2023 Jun 30;102(26):e34166. doi: 10.1097/MD.0000000000034166 (PMC10313258; doi:10.1097/MD.0000000000034166)
Supplement: Supplementary file 1 [file medi-102-e34166-s001.pdf]

Supplementary Table 1 GO enrichment result of HF DEGs

ONTOLOGY	ID	Description	pvalue	Count
BP	GO:0015701	bicarbonate transport	9.65E-06	5
BP	GO:0030198	extracellular matrix organization	6.66E-05	11
BP	GO:0043062	extracellular structure organization	6.81E-05	11
BP	GO:0045229	external encapsulating structure organization	7.13E-05	11
BP	GO:1990748	cellular detoxification	0.000122447	6
BP	GO:0015671	oxygen transport	0.000124883	3
CC	GO:0062023	collagen-containing extracellular matrix	7.03E-11	19
CC	GO:0071682	endocytic vesicle lumen	8.90E-06	4
CC	GO:0031838	haptoglobin-hemoglobin complex	4.79E-05	3
CC	GO:0005833	hemoglobin complex	6.36E-05	3
CC	GO:0005604	basement membrane	0.000444445	5
MF	GO:0005201	extracellular matrix structural constituent	2.55E-07	10
MF	GO:0030021	extracellular matrix structural constituent conferring compression resistance	3.26E-07	5
MF	GO:0031720	haptoglobin binding	3.58E-05	3
MF	GO:0005344	oxygen carrier activity	0.000106551	3
MF	GO:0019825	oxygen binding	0.000140715	4
MF	GO:0005125	cytokine activity	0.000209699	8
MF	GO:0016209	antioxidant activity	0.000304339	5
MF	GO:0008009	chemokine activity	0.000343918	4
MF	GO:0004601	peroxidase activity	0.000465439	4
MF	GO:0048018	receptor ligand activity	0.000496205	11
MF	GO:0030546	signaling receptor activator activity	0.000549525	11
MF	GO:0016684	oxidoreductase activity, acting on peroxide as acceptor	0.000614825	4
MF	GO:0004435	phosphatidylinositol phospholipase C activity	0.000716939	3
MF	GO:0004629	phospholipase C activity	0.000894411	3
MF	GO:0005518	collagen binding	0.001196611	4
MF	GO:0042379	chemokine receptor binding	0.001263621	4
MF	GO:1901681	sulfur compound binding	0.00204955	7
MF	GO:0016829	lyase activity	0.002149285	6

MF	GO:0050660	flavin adenine dinucleotide binding	0.002284074	4
MF	GO:0030246	carbohydrate binding	0.002379226	7
MF	GO:0005126	cytokine receptor binding	0.002532438	7
MF	GO:0016903	oxidoreductase activity, acting on the aldehyde or oxo group of donors	0.00334029	3
MF	GO:0016840	carbon-nitrogen lyase activity	0.003423231	2
MF	GO:0001664	G protein-coupled receptor binding	0.00368684	7
MF	GO:0004089	carbonate dehydratase activity	0.003976011	2
