# Supplementary material for: Identifying immune cell infiltration and diagnostic biomarkers in heart failure and osteoarthritis by bioinformatics analysis
Source: Medicine (Baltimore). 2023 Jun 30;102(26):e34166. doi: 10.1097/MD.0000000000034166 (PMC10313258; doi:10.1097/MD.0000000000034166)
Supplement: Supplementary file 2 [file medi-102-e34166-s002.pdf]

Supplementary Table 2 KEGG enrichment of HF upregulated DEGs

ID	Description	pvalue	Count
hsa05143	African trypanosomiasis	3.24E-05	4
hsa05144	Malaria	0.000107813	4
hsa04024	cAMP signaling pathway	0.00075868	6
hsa04933	AGE-RAGE signaling pathway in diabetic complications	0.001531048	4
hsa05310	Asthma	0.010489909	2
hsa05323	Rheumatoid arthritis	0.011241146	3
hsa04925	Aldosterone synthesis and secretion	0.012949559	3
hsa04061	Viral protein interaction with cytokine and cytokine receptor	0.013672374	3
hsa04060	Cytokine-cytokine receptor interaction	0.015543285	5
hsa05332	Graft-versus-host disease	0.018758698	2
hsa05166	Human T-cell leukemia virus 1 infection	0.024767051	4
hsa04340	Hedgehog signaling pathway	0.032103822	2
hsa04072	Phospholipase D signaling pathway	0.037944095	3
hsa05321	Inflammatory bowel disease	0.042152896	2
