# Supplementary material for: Identifying immune cell infiltration and diagnostic biomarkers in heart failure and osteoarthritis by bioinformatics analysis
Source: Medicine (Baltimore). 2023 Jun 30;102(26):e34166. doi: 10.1097/MD.0000000000034166 (PMC10313258; doi:10.1097/MD.0000000000034166)
Supplement: Supplementary file 3 [file medi-102-e34166-s003.pdf]

Supplementary Table 3 KEGG enrichment of HF downregulated DEGs

**ID**	**Description**	**pvalue**	**Count**
hsa00982	Drug metabolism - cytochrome P450	9.80E-05	4
hsa05310	Asthma	0.004986	2
hsa00350	Tyrosine metabolism	0.006685	2
hsa00760	Nicotinate and nicotinamide metabolism	0.006685	2
hsa00260	Glycine, serine and threonine metabolism	0.008206	2
hsa04975	Fat digestion and absorption	0.00944	2
hsa00280	Valine, leucine and isoleucine degradation	0.011668	2
hsa00480	Glutathione metabolism	0.016742	2
hsa00830	Retinol metabolism	0.02259	2
hsa00980	Metabolism of xenobiotics by cytochrome P450	0.02916	2
hsa05130	Pathogenic Escherichia coli infection	0.029306	3
hsa04540	Gap junction	0.036398	2
hsa00130	Ubiquinone and other terpenoid-quinone biosynthesis	0.037207	1
hsa04657	IL-17 signaling pathway	0.041042	2
hsa00564	Glycerophospholipid metabolism	0.044256	2
hsa04061	Viral protein interaction with cytokine and cytokine receptor	0.045898	2
hsa04972	Pancreatic secretion	0.047562	2
