# Supplementary material for: Identifying immune cell infiltration and diagnostic biomarkers in heart failure and osteoarthritis by bioinformatics analysis
Source: Medicine (Baltimore). 2023 Jun 30;102(26):e34166. doi: 10.1097/MD.0000000000034166 (PMC10313258; doi:10.1097/MD.0000000000034166)
Supplement: Supplementary file 4 [file medi-102-e34166-s004.pdf]

Supplementary Table 4 GO enrichment of OA DEGs

ONTOLOGY	ID	Description	pvalue	Count
BP	GO:0098883	synapse pruning	2.30E-06	4
BP	GO:0007162	negative regulation of cell adhesion	4.19E-06	13
BP	GO:0031669	cellular response to nutrient levels	4.61E-06	11
BP	GO:0031667	response to nutrient levels	5.30E-06	16
BP	GO:0051098	regulation of binding	6.82E-06	14
BP	GO:0002697	regulation of immune effector process	7.78E-06	16
BP	GO:0071295	cellular response to vitamin	7.88E-06	5
BP	GO:0009991	response to extracellular stimulus	1.07E-05	16
BP	GO:0031668	cellular response to extracellular stimulus	1.34E-05	11
BP	GO:0001501	skeletal system development	1.35E-05	16
BP	GO:0051928	positive regulation of calcium ion transport	1.50E-05	8
BP	GO:0043393	regulation of protein binding	1.59E-05	10
BP	GO:0060333	interferon-gamma-mediated signaling pathway	2.29E-05	7
BP	GO:0002685	regulation of leukocyte migration	2.34E-05	10
BP	GO:0001503	ossification	2.50E-05	14
BP	GO:0071496	cellular response to external stimulus	2.86E-05	12
BP	GO:0070374	positive regulation of ERK1 and ERK2 cascade	2.88E-05	10
BP	GO:0150146	cell junction disassembly	3.16E-05	4
BP	GO:0051099	positive regulation of binding	3.18E-05	9
BP	GO:0001659	temperature homeostasis	3.33E-05	9
BP	GO:0031670	cellular response to nutrient	4.30E-05	5
BP	GO:0071346	cellular response to interferon-gamma	4.37E-05	9
BP	GO:0106106	cold-induced thermogenesis	6.27E-05	8
BP	GO:0120161	regulation of cold-induced thermogenesis	6.27E-05	8
BP	GO:0070482	response to oxygen levels	6.89E-05	13
BP	GO:0030198	extracellular matrix organization	8.48E-05	13
BP	GO:0043062	extracellular structure organization	8.70E-05	13
BP	GO:0045229	external encapsulating structure organization	9.15E-05	13
BP	GO:1990830	cellular response to leukemia inhibitory factor	9.59E-05	6
BP	GO:1990845	adaptive thermogenesis	9.61E-05	8
BP	GO:0034341	response to interferon-gamma	9.97E-05	9
BP	GO:1990823	response to leukemia inhibitory factor	0.000103	6
BP	GO:0002455	humoral immune response mediated by circulating immunoglobulin	0.00011	8
BP	GO:0032092	positive regulation of protein binding	0.000126	6
BP	GO:0070372	regulation of ERK1 and ERK2 cascade	0.000127	11
BP	GO:0002688	regulation of leukocyte chemotaxis	0.000127	7

BP	GO:0050808	synapse organization	0.000136	13
BP	GO:0042476	odontogenesis	0.000156	7
BP	GO:0043380	regulation of memory T cell differentiation	0.000165	3
BP	GO:0002460	adaptive immune response based on somatic recombination of immune receptors built from immunoglobulin superfamily domains	0.000177	12
BP	GO:0033273	response to vitamin	0.000186	6
BP	GO:1903035	negative regulation of response to wounding	0.000197	6
BP	GO:0002923	regulation of humoral immune response mediated by circulating immunoglobulin	0.000213	3
BP	GO:0016322	neuron remodeling	0.000213	3
BP	GO:0043379	memory T cell differentiation	0.000213	3
BP	GO:0097529	myeloid leukocyte migration	0.000214	9
BP	GO:0070371	ERK1 and ERK2 cascade	0.000216	11
BP	GO:0071900	regulation of protein serine/threonine kinase activity	0.00022	14
BP	GO:1901216	positive regulation of neuron death	0.000223	6
BP	GO:0090715	immunological memory formation process	0.00027	3
BP	GO:0033280	response to vitamin D	0.000273	4
BP	GO:0030595	leukocyte chemotaxis	0.000279	9
BP	GO:0048545	response to steroid hormone	0.000281	11
BP	GO:0120162	positive regulation of cold-induced thermogenesis	0.000297	6
BP	GO:0002920	regulation of humoral immune response	0.000317	7
BP	GO:0002468	dendritic cell antigen processing and presentation	0.000335	3
BP	GO:0032102	negative regulation of response to external stimulus	0.000339	12
BP	GO:0055074	calcium ion homeostasis	0.000348	13
BP	GO:0002696	positive regulation of leukocyte activation	0.000397	12
BP	GO:0051924	regulation of calcium ion transport	0.000408	9
BP	GO:0061387	regulation of extent of cell growth	0.000432	6
BP	GO:0001666	response to hypoxia	0.00044	11
BP	GO:0031099	regeneration	0.000452	8
BP	GO:0060348	bone development	0.000468	8
BP	GO:0043116	negative regulation of vascular permeability	0.000493	3
BP	GO:0090713	immunological memory process	0.000493	3
BP	GO:0050867	positive regulation of cell activation	0.000505	12
BP	GO:0042594	response to starvation	0.000518	8
BP	GO:0010959	regulation of metal ion transport	0.000534	9

BP	GO:0032963	collagen metabolic process	0.000555	6
BP	GO:0031960	response to corticosteroid	0.000568	7
BP	GO:0002449	lymphocyte mediated immunity	0.000583	11
BP	GO:0036293	response to decreased oxygen levels	0.000583	11
BP	GO:0007568	aging	0.000588	10
BP	GO:0071675	regulation of mononuclear cell migration	0.000611	6
BP	GO:1902107	positive regulation of leukocyte differentiation	0.000614	7
BP	GO:1903708	positive regulation of hemopoiesis	0.000614	7
BP	GO:0060326	cell chemotaxis	0.000618	10
BP	GO:0043114	regulation of vascular permeability	0.000622	4
BP	GO:0061045	negative regulation of wound healing	0.00064	5
BP	GO:0060284	regulation of cell development	0.000647	13
BP	GO:0050727	regulation of inflammatory response	0.000668	11
BP	GO:0002573	myeloid leukocyte differentiation	0.000674	8
BP	GO:0044706	multi-multicellular organism process	0.000674	8
BP	GO:0002483	antigen processing and presentation of endogenous peptide antigen	0.000693	3
BP	GO:0031214	biomineral tissue development	0.00077	7
BP	GO:0002703	regulation of leukocyte mediated immunity	0.000789	8
BP	GO:0007626	locomotory behavior	0.000799	7
BP	GO:0042551	neuron maturation	0.00081	4
BP	GO:0090280	positive regulation of calcium ion import	0.00081	3
BP	GO:0110148	biomineralization	0.000828	7
BP	GO:0072507	divalent inorganic cation homeostasis	0.000841	13
BP	GO:0022407	regulation of cell-cell adhesion	0.000847	12
BP	GO:0050870	positive regulation of T cell activation	0.000865	8
BP	GO:0007584	response to nutrient	0.000922	7
BP	GO:0006874	cellular calcium ion homeostasis	0.000934	12
BP	GO:0030574	collagen catabolic process	0.000956	4
BP	GO:0050863	regulation of T cell activation	0.001026	10
BP	GO:0001913	T cell mediated cytotoxicity	0.001035	4
BP	GO:0030195	negative regulation of blood coagulation	0.001035	4
BP	GO:0002699	positive regulation of immune effector process	0.001067	8
BP	GO:0071305	cellular response to vitamin D	0.001079	3
BP	GO:0002819	regulation of adaptive immune response	0.001098	7
BP	GO:1903037	regulation of leukocyte cell-cell adhesion	0.001099	10
BP	GO:1900047	negative regulation of hemostasis	0.001119	4
BP	GO:0002705	positive regulation of leukocyte mediated immunity	0.001137	6
BP	GO:0032355	response to estradiol	0.001137	6
BP	GO:0050890	cognition	0.001171	9

BP	GO:0001649	osteoblast differentiation	0.001198	8
BP	GO:0016064	immunoglobulin mediated immune response	0.001198	8
BP	GO:0007566	embryo implantation	0.001207	4
BP	GO:0001964	startle response	0.001233	3
BP	GO:1902105	regulation of leukocyte differentiation	0.001262	9
BP	GO:0060560	developmental growth involved in morphogenesis	0.001268	8
BP	GO:1904645	response to amyloid-beta	0.001301	4
BP	GO:0019724	B cell mediated immunity	0.001304	8
BP	GO:0007565	female pregnancy	0.001386	7
BP	GO:0046683	response to organophosphorus	0.001392	6
BP	GO:0043113	receptor clustering	0.001399	4
BP	GO:0050819	negative regulation of coagulation	0.001399	4
BP	GO:0002683	negative regulation of immune system process	0.001455	11
BP	GO:0051480	regulation of cytosolic calcium ion concentration	0.001499	10
BP	GO:0002687	positive regulation of leukocyte migration	0.001564	6
BP	GO:1903039	positive regulation of leukocyte cell-cell adhesion	0.001625	8
BP	GO:0043123	positive regulation of I-kappaB kinase/NF-kappaB signaling	0.001626	7
BP	GO:0002690	positive regulation of leukocyte chemotaxis	0.001627	5
BP	GO:0006816	calcium ion transport	0.001635	11
BP	GO:0007611	learning or memory	0.001669	8
BP	GO:0001912	positive regulation of leukocyte mediated cytotoxicity	0.001724	4
BP	GO:0045598	regulation of fat cell differentiation	0.001752	6
BP	GO:0019883	antigen processing and presentation of endogenous antigen	0.001773	3
BP	GO:0051591	response to cAMP	0.001791	5
BP	GO:0043388	positive regulation of DNA binding	0.001844	4
BP	GO:0072503	cellular divalent inorganic cation homeostasis	0.001876	12
BP	GO:0051251	positive regulation of lymphocyte activation	0.001928	10
BP	GO:0045672	positive regulation of osteoclast differentiation	0.001981	3
BP	GO:1901890	positive regulation of cell junction assembly	0.00206	5
BP	GO:0071674	mononuclear cell migration	0.002076	7
BP	GO:0051960	regulation of nervous system development	0.002088	11

BP	GO:0002763	positive regulation of myeloid leukocyte differentiation	0.002099	4
BP	GO:0031102	neuron projection regeneration	0.002099	4
BP	GO:0001933	negative regulation of protein phosphorylation	0.002178	10
BP	GO:0045785	positive regulation of cell adhesion	0.002206	11
BP	GO:0051055	negative regulation of lipid biosynthetic process	0.002236	4
CC	GO:0062023	collagen-containing extracellular matrix	6.62E-11	22
MF	GO:0005201	extracellular matrix structural constituent	5.25E-06	10
