# Supplementary material for: Identifying immune cell infiltration and diagnostic biomarkers in heart failure and osteoarthritis by bioinformatics analysis
Source: Medicine (Baltimore). 2023 Jun 30;102(26):e34166. doi: 10.1097/MD.0000000000034166 (PMC10313258; doi:10.1097/MD.0000000000034166)
Supplement: Supplementary file 5 [file medi-102-e34166-s005.pdf]

Supplementary Table 5 KEGG enrichment of OA upregulated DEGs

ID	Description	pvalue	Count
hsa04610	Complement and coagulation cascades	2.32E-05	6
hsa05150	Staphylococcus aureus infection	4.63E-05	6
hsa05322	Systemic lupus erythematosus	0.000318088	6
hsa04512	ECM-receptor interaction	0.000325647	5
hsa05323	Rheumatoid arthritis	0.003723331	4
hsa04060	Cytokine-cytokine receptor interaction	0.003860582	7
hsa04672	Intestinal immune network for IgA production	0.004539515	3
hsa04640	Hematopoietic cell lineage	0.004656747	4
hsa04979	Cholesterol metabolism	0.004805946	3
hsa04061	Viral protein interaction with cytokine and cytokine receptor	0.004826282	4
hsa04064	NF-kappa B signaling pathway	0.005546008	4
hsa04310	Wnt signaling pathway	0.005617251	5
hsa04151	PI3K-Akt signaling pathway	0.010244656	7
hsa04380	Osteoclast differentiation	0.011398456	4
hsa04926	Relaxin signaling pathway	0.011704067	4
hsa04510	Focal adhesion	0.012022129	5
hsa05205	Proteoglycans in cancer	0.013008413	5
hsa05133	Pertussis	0.01520549	3
hsa05140	Leishmaniasis	0.015746051	3
hsa04612	Antigen processing and presentation	0.01629738	3
hsa05310	Asthma	0.019016849	2
hsa04145	Phagosome	0.020240097	4
hsa05330	Allograft rejection	0.027885033	2
hsa05146	Amoebiasis	0.032788424	3
hsa05332	Graft-versus-host disease	0.033562704	2
hsa04940	Type I diabetes mellitus	0.035047205	2
hsa05202	Transcriptional misregulation in cancer	0.043218989	4
hsa04670	Leukocyte transendothelial migration	0.043367861	3
