# Supplementary material for: Identifying immune cell infiltration and diagnostic biomarkers in heart failure and osteoarthritis by bioinformatics analysis
Source: Medicine (Baltimore). 2023 Jun 30;102(26):e34166. doi: 10.1097/MD.0000000000034166 (PMC10313258; doi:10.1097/MD.0000000000034166)
Supplement: Supplementary file 6 [file medi-102-e34166-s006.pdf]

Supplementary Table 6 KEGG enrichment of OA downregulated DEGs

ID	Description	pvalue	Count
hsa04964	Proximal tubule bicarbonate reclamation	0.006425	2
hsa05216	Thyroid cancer	0.016142	2
hsa05219	Bladder cancer	0.019612	2
hsa00071	Fatty acid degradation	0.021454	2
hsa04110	Cell cycle	0.02849	3
hsa04068	FoxO signaling pathway	0.031474	3
hsa05213	Endometrial cancer	0.037371	2
hsa05224	Breast cancer	0.042084	3
hsa05217	Basal cell carcinoma	0.043432	2
hsa00010	Glycolysis / Gluconeogenesis	0.048526	2
hsa05221	Acute myeloid leukemia	0.048526	2
